# Re‐establishing indwelling pleural catheter patency with alteplase after failure of streptokinase

**DOI:** 10.1002/rcr2.639

**Published:** 2020-08-06

**Authors:** Mohamed Faisal, Siti Nurhanis, Nik Nuratiqah Nik Abeed, Boon Hau Ng, Andrea Yu‐Lin Ban

**Affiliations:** ^1^ Respiratory Unit, Department of Medicine, Faculty of Medicine Universiti Kebangsaan Malaysia Medical Centre Kuala Lumpur Malaysia

**Keywords:** Alteplase, blocked, indwelling pleural catheter, malignant pleural effusion, streptokinase

## Abstract

Indwelling pleural catheter (IPC) has revolutionized the management of malignant pleural effusion (MPE). IPC is relatively safe, although complications can occur. We report a 53‐year‐old woman with stage IVA lung adenocarcinoma and recurrent MPE. Two months post insertion, the IPC was blocked with residual effusion and presence of new loculations. Attempts to restore patency with six doses of intrapleural (IP) streptokinase failed. She was referred to our centre for further management. We used a single dose of 2.5 mg IP alteplase which was successful in establishing patency of the IPC and draining the effusion. This case highlights the safety and efficacy of IP alteplase via IPC following a failed instillation of streptokinase.

## Graphical abstract

We describe the successful use of a single low‐dose intrapleural (IP) alteplase in both indwelling pleural catheter blockage and symptomatic loculation drainage, following a failed therapy with six doses of IP streptokinase.

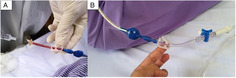

## Introduction

Indwelling pleural catheter (IPC) is a multi‐fenestrated silicone tube tunnelled subcutaneously with a one‐way valve allowing ambulatory drainage of pleural effusion. IPC is used mainly in patients with recurrent malignant pleural effusion (MPE); however, it can also be used in non‐malignant effusions such as hepatic hydrothorax, chronic heart failure, or chylothorax [1]. Following IPC insertion, symptomatic loculations may be present in up to 14% and as early as two months [1]. Management of these loculations include intrapleural (IP) fibrinolytics (with/without dornase alfa) or placement of IPC in a different locule [2]. We describe the successful use of a single low‐dose IP alteplase in both IPC blockage and symptomatic loculation drainage, following failed therapy with six doses of IP streptokinase.

## Case Report

**Figure 1:**
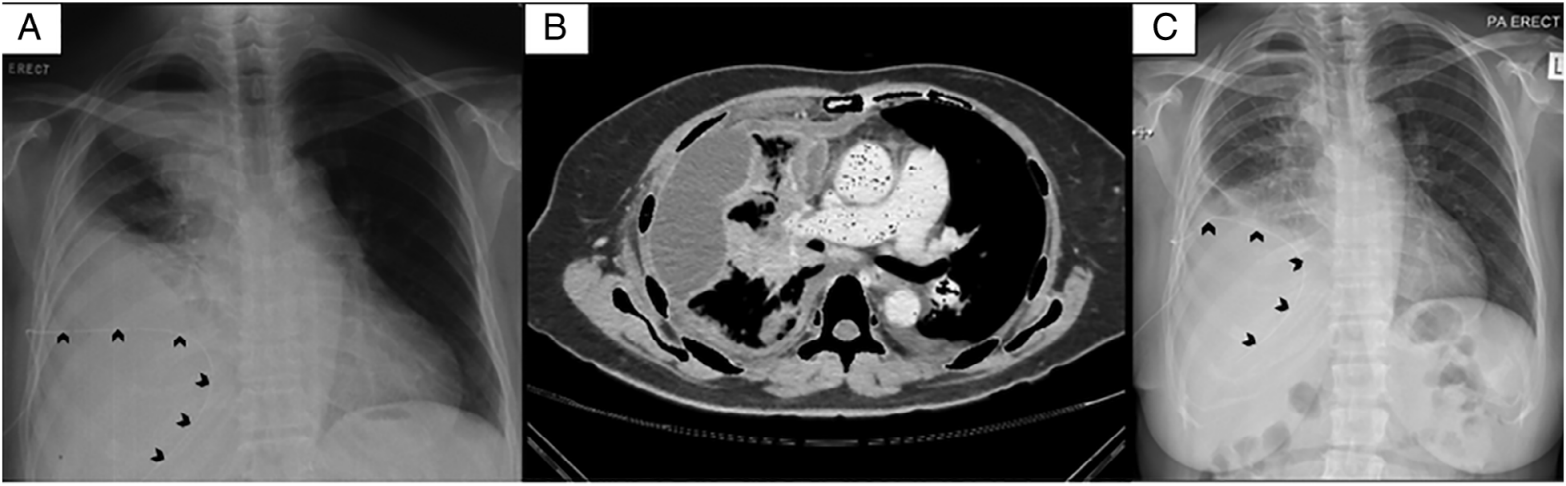
Chest radiograph (A) showed a loculated right pleural effusion with indwelling pleural catheter (IPC) in situ (black arrows). Computed tomography (CT) of the thorax (B) showed a loculated right pleural effusion. Chest radiograph (C) post IP alteplase showed improvement with minimal residual pleural effusion and IPC in situ (black arrows) with elevated right hemidiaphragm.

A 53‐year‐old woman with stage IVA (T2bN3M1a) lung adenocarcinoma with negative epidermal growth factor receptor (EGFR) driver mutation presented with a massive right pleural effusion. Pleural fluid cytology confirmed metastatic adenocarcinoma and thyroid transcriptase factor 1 (TTF‐1) was positive from immunohistochemistry. IPC (Rocket® IPC, Rocket Medical, Washington, UK) was inserted and complicated by poor drainage at two months which did not resolve with six doses of IP streptokinase (500,000 IU per instillation). Chest radiograph showed loculated right pleural effusion (Fig. 1A). Contrast‐enhanced computed tomography (CECT) of the thorax post fibrinolytic therapy showed multiloculated right pleural effusion with the largest locule at the right anterolateral mid hemithorax (Fig. 1B) with the tip of IPC seen at the posterior lower right thorax. The patient was referred to our centre for further management.

**Figure 2:**
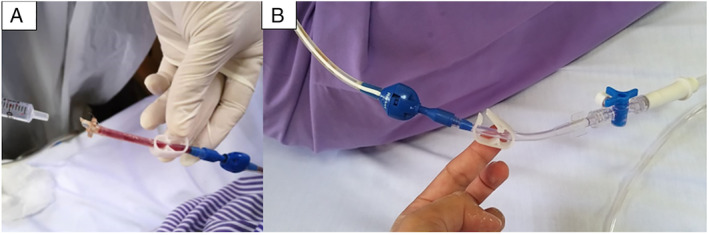
Damaged tubing (A) attached to the universal indwelling pleural catheter (IPC). Replacement with a new functioning Rocket® IPC adaptor (B).

Upon arrival to our hospital, she was a bit distressed and mildly breathless with respiratory rate of 24/min. We performed a bedside thoracic sonography which confirmed a multiloculated effusion at the right upper lateral and lower posterior chest. We also found the tubing attached to the universal IPC adaptor to be damaged (Fig. 2A). We replaced this with a new functioning Rocket® IPC adaptor (Fig. 2B) and proceeded to manually flush and aspirate 50 cc of haemoserous fluid. She was afebrile throughout with no evidence of infection clinically. The pleural fluid culture was negative. We instilled 2.5 mg of alteplase which was diluted with 50 mL NaCl through the IPC. The IPC was clamped for 45 min and then opened. We drained 500 mL haemoserous pleural fluid over 6 h. Repeated chest radiograph post IP alteplase (Fig. 1C) and bedside thoracic sonography showed resolution of effusion with elevated right hemidiaphragm. Her dyspnoea was relieved, she was discharged well, and continued drainage at home.

## Discussion

MPE is common with a reported incidence of over 150,000 cases in the United States annually [3]. Symptoms of MPE range from asymptomatic to symptoms of breathlessness, orthopnoea, reduced effort tolerance, and reduced quality of life. The goal of treatment is alleviating these symptoms. This can be achieved with thoracentesis per needed basis for immediate relieve, chemical pleurodesis via intercostal chest tube or pleuroscopy, IPC insertion with/without pleurodesis, cancer‐specific therapy with chemotherapy/radiotherapy, and surgery [3, 4]. The potential advantage of IPC over pleurodesis is that it can be used in non‐expandable lungs.

Blockages of some of IPC fenestration can occur due to inflammatory debris from pleural inflammation. However, incidence of complete occlusion is <5% and management includes saline flushing and manipulation along the catheter [1]. These inflammatory process can also induce septations and pleural loculation. In IPC‐treated patients, symptomatic loculations are reported to be around 5–14% [1]. IP fibrinolytics is a feasible treatment option in these situations.

The success rate of IP streptokinase in loculated pleural effusion has been reported at 72% [5]. Our patient had cessation of drainage and symptomatic pleural loculation which failed to respond to six doses of IP streptokinase. We successfully drained the loculation with instillation of a single dose of alteplase in our centre, with resulting improvement both clinically and radiologically.

Pleural infections related to IPC have been reported to be around 4.8% [1]. It typically occurs six to eight weeks post insertion. While the exact pathophysiology is unknown, postulations include migration from bacteria colonizing the skin to the pleura along the IPC and entry of bacteria from the infected lung into the pleural cavity. The mainstay of treatment is antibiotics; however, removal of IPC or IP fibrinolytics may be needed if treatment fails [1].

The decision for alteplase was in line with a recent modified Delphi consensus which recommended the use of alteplase in such situations [2]. The recommended dosage of alteplase is still not well established. We decided to use a lower dose of 2.5 mg to prevent the risk of pleural or systemic bleeding as the patient had already received multiple doses of streptokinase in the previous centre. This single dose proved successful to restore IPC patency. As there was no evidence of pleural infection, we did not add IP dornase alfa (Pulmozyme, Hoffmann‐La Roche Ltd).

The development of anti‐streptokinase antibody following IP instillation has been reported [5]. The presence of these antibodies may lead to reduced effectiveness of future thrombolysis and sensitization and subsequent allergic reaction if used for systemic thrombolysis. All these factors generally favour the use of other agents as an antifibrinolytic agent.

In summary, IPC is a good treatment option in MPE. Alteplase is effective and safe in a blocked IPC with/without pleural loculation. Clinicians who provide IPC service should be able to anticipate and manage IPC complications as there is growing interest in the use of IPC in MPE due to its therapeutic benefits.

### Disclosure Statement

Appropriate written informed consent was obtained for publication of this case report and accompanying images.

This patient was part of a study entitled “A 8 Year Review of Operational Practice and Clinical Outcome of Complex Pleural Effusion Before and After the Advent of Intrapleural Alteplase” approved by the UKM Ethics Committee (FF‐2020‐143).
